# Machine Learning and Precision Medicine in Emergency Medicine: The Basics

**DOI:** 10.7759/cureus.17636

**Published:** 2021-09-01

**Authors:** Sangil Lee, Samuel H Lam, Thiago Augusto Hernandes Rocha, Ross J Fleischman, Catherine A Staton, Richard Taylor, Alexander T Limkakeng

**Affiliations:** 1 Emergency Medicine, University of Iowa Carver College of Medicine, Iowa City, USA; 2 Emergency Medicine, Sutter Medical Center, Sacramento, USA; 3 Division of Emergency Medicine, Department of Surgery, Duke University School of Medicine, Durham, USA; 4 Emergency Medicine, Harbor-UCLA Medical Center, Los Angeles, USA; 5 Department of Emergency Medicine, Yale University, New Haven, USA

**Keywords:** machine learning, artificial intelligence, precision medicine, research in emergency medicine, risk prediction

## Abstract

As machine learning (ML) and precision medicine become more readily available and used in practice, emergency physicians must understand the potential advantages and limitations of the technology. This narrative review focuses on the key components of machine learning, artificial intelligence, and precision medicine in emergency medicine (EM). Based on the content expertise, we identified articles from EM literature. The authors provided a narrative summary of each piece of literature. Next, the authors provided an introduction of the concepts of ML, artificial intelligence as an extension of ML, and precision medicine. This was followed by concrete examples of their applications in practice and research. Subsequently, we shared our thoughts on how to consume the existing research in these subjects and conduct high-quality research for academic emergency medicine. We foresee that the EM community will continue to adapt machine learning, artificial intelligence, and precision medicine in research and practice. We described several key components using our expertise.

## Introduction and background

Definition 

Artificial intelligence (AI) is the theory and development of computer systems that are able to perform tasks that normally require human intelligence such as visual perception, speech recognition, decision-making, and translation between languages [1-2]. Over the last decade, coinciding with the explosion of data in medicine and the ubiquity of electronic health records (EHRs), there has been growing interest in AI within emergency medicine (EM) [3]. These developments have also been spurred on by the rapid increase in the storage capacity and processing power of computers [4]. A key component of AI is machine learning (ML), where machines are trained on available data to learn a specific task (e.g., predicting whether a patient has sepsis). ML is now used in diverse areas of medicine, including radiology, dermatology, and ophthalmology. Precision medicine is another extension where ML can be used as part of algorithms, as described further in this review. A key distinction between ML predictive models and traditional modeling methods is that ML is less “supervised,” that is, that the computer is able to use a wider range and larger number of variables to identify relationships as opposed to a limited set of variables identified before an investigator defines and evaluates them.

Although there are multiple prior papers using ML and AI in EM, including some recent reviews of the topic, few papers describe the basic concepts in a digestible format for emergency physicians without prior statistical or research backgrounds [5]. In this paper, we provide an introduction to the concepts of ML and precision medicine. This is followed by concrete examples of their applications in practice and research in EM. Subsequently, we share our thoughts on how to consume the existing research in these subjects and conduct high-quality research for the academic EM community.

Terminology

Since terminology in ML may not be a common language to the EM community, we listed several key terminologies in Table 1.

**Table 1:** Terminology unique to machine learning literature

Terms	Explanation
Machine learning [1]	Algorithms that continually improve their functioning (learning) based on exposure to data
Deep learning [2]	Neural networks that have multiple hidden (deep) layers to more effectively represent complex relationships
Natural language processing [2]	Use of computer algorithms for processing text documents (e.g. provider notes)
Precision medicine [4]	Medical care designed to optimize efficiency or therapeutic benefit for particular groups of patients, especially by using genetic or molecular profiling
Supervised learning [2]	A subfield of machine learning where computer algorithms are given data with a known output of interest (e.g., patients who did or did not have pulmonary emboli) and a model is developed to predict that output from potential inputs (e.g., vital signs, risk factors, exam findings, etc.)
Unsupervised Learning [2]	A subfield of machine learning where computer algorithms are given data without a single pre-specified output but instead intrinsic patterns or relationships of interest (e.g., clustering)

Basic steps of AI in healthcare

ML and AI can, at times, require heavy computational resources that can be an obstacle to the junior-level researcher who is interested in these areas. Often, though, this type of resource is available by inter-departmental or inter-campus collaboration, and in general, AI and ML activities should be viewed more from a team perspective. We outline some basic steps in the AI healthcare pipeline below.

Data Sources and Extraction

AI research in EM is based largely on data acquired from the EHR [6]. As institutions have increasingly invested in data collection processes, infrastructure, database storage methods, and distributed computing systems, data acquisition from the EHR has accelerated. Data collected from EHRs can include basic information such as patient demographics, diagnoses, labs, and medications to operation-related metrics, such as census, length of stay, and patient satisfaction scores, to even unstructured data like clinical notes through the use of natural language processing [2,7-9].

Data Preprocessing 

Data preprocessing, or wrangling, is a critical step in AI development in which data are formatted and modified for further downstream use in AI algorithms. Modifications can include changing continuous values into categorical values, exploring data for outliers and removing them, and imputing missing values [10].

Algorithmic Development

The algorithm development can use several methods, but the developer selects a combination of algorithms to address the prediction of interest. The sample can be split into a training set and testing set or use k-fold cross-validation so that the algorithm can be applied to the training set and tested for validation. By conducting model selection independently in each trial of the model fitting procedure, the risk of overfitting during hyperparameter tuning is reduced [2]. The model performance is evaluated using the predictive ability, for example, area under the curve (AUC). 

Decision Support, ML, and Interpretability-the Doctor-AI Relationship

Because EM as a field is focused on diagnosis and disposition, EM researchers have developed numerous decision aides, or rules, such as the pulmonary embolism rule-out criteria (PERC) and HEART score to aid healthcare providers [11-15].

While the decision rules are helpful, they are often limited to a small number of data points and do not factor in complex relationships between variables. AI incorporated into EHR clinical decision support systems (CDSS), with the ability to factor in hundreds of variables and their interactions, holds promise as a better method to aid providers.

Though it is an attractive option, ML raises an issue of interpretability - a critical concept that patients and clinicians rely on to convey information. If data analysis stays in a black box, clinicians may hesitate to follow the ML predictions. While newer methods to improve interpretability exist, there will likely always be some tradeoff. We list a comparison of clinical decision rules and ML models in Table 2 and common ML models by interpretability, computation time, and a number of predictors in Table 3. While EM providers are familiar with using clinical decision rules as a part of medical decision-making, the use of ML in this context has not been standardized in the EM community. 

**Table 2:** Comparison of conventional clinical decision rule vs machine-learning model PERC rule: Pulmonary Embolism Rule Out Criteria, ECG: Electrocardiogram

	Clinical Decision Rule	Machine Learning Model
Complexity	Simple	Complex
Interpretability	Easy	Easy to difficult
Operator	Human	Computer
Clinical application (example)	PERC rule	ECG interpretation

**Table 3:** Type of machine learning algorithms and their characteristics Source: [2]

Model	Interpretable	Computation time	Allows predictor > sample size*
Linear Regression	Yes	Low	No
Logistic Regression	Yes	Low	No
Partial Least Squares	Yes	Low	Yes
Ridge Regression	Yes	Low	No
Lasso/Elastic Net	Yes	Low	Yes
Classification and Regression Trees	Possible	Low	Yes
Linear Discriminant Analysis	Possible	Low	No
Multivariate Adaptive Regression Spline	Possible	Intermediate	Yes
C5.0 Decision Trees	Possible	High	Yes
K-Nearest Neighbors	No	Low	Yes
Naive Bayes	No	Intermediate	Yes
Support Vector Machine	No	High	Yes
Neural Networks	No	High	Yes
Random Forest	No	High	Yes

## Review

Database search strategy

Our authors chose articles based on the selected areas of expertise in the intersection of AI and EM to write this narrative review. Each author searched and identified the literature in their area of expertise.

Application of ML and precision medicine in EM research

This review will present a short summary of selected examples of ML and precision medicine in EM research and innovation. 

AI in medical imaging

The use of AI in medical imaging has many potential advantages. It could enable clinicians to make timely diagnoses and lighten the cognitive load by preliminary processing for optimal review, detection, and flagging of abnormal studies, and focusing on high-yield areas. It could assist in generating differential diagnoses to ensure that rare (but clinically important) diagnoses are considered in image interpretation. Furthermore, it could allow quality care to be delivered in rural and remote locations by assisting in the acquisition and interpretation of critical images.

**Figure 1:**
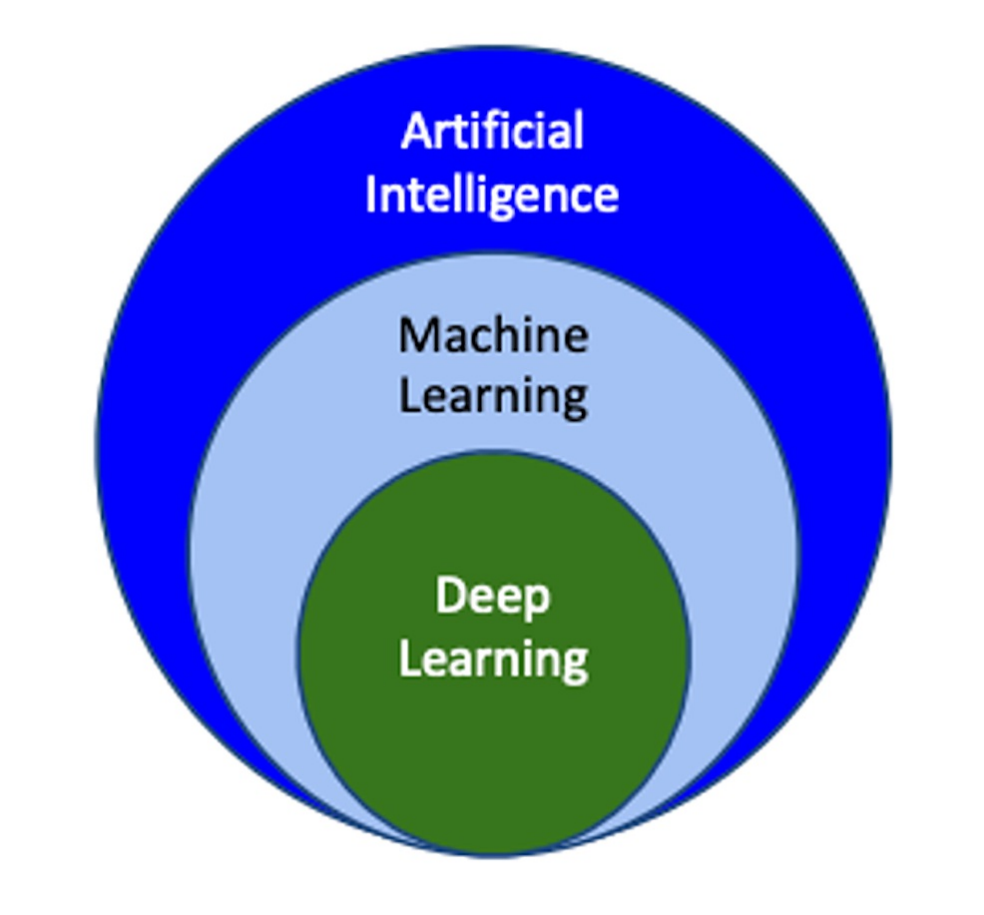
The framework of deep learning, machine learning, and artificial intelligence

**Figure 2:**
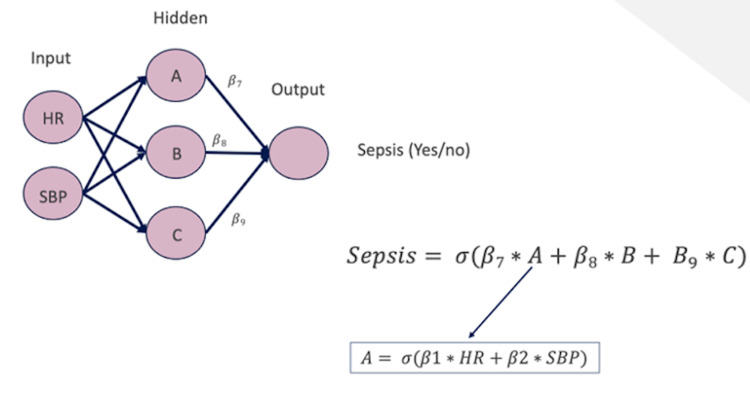
Diagram showing neural network to predict sepsis

Much of the current research on AI in medical imaging focuses on the use of deep learning (DL) to assist in pattern recognition and quantitative measurement [2]. DL refers to the branch of ML utilizing neural networks for unsupervised reinforcement learning (Figure 1). Neural networks are composed of layers of nodes or “neurons;” each performs a computational operation through which information flows by means of weighted interconnections. Each node processes information from the prior layer and forwards its output “feature” to nodes in the next layer, eventually culminating in a final output (Figure 2). During DL training, weights and biases are learned and fine-tuned. A common neural network utilized for DL in medical imaging is a convolutional neural network (CNN). 

Examples of DL advances in EM radiology relevant to the practice of EM include stroke diagnosis [16], pulmonary embolism diagnosis [17], detection of intracranial hemorrhages [17], fracture diagnosis [17], predicting cerebral aneurysm rupture risk [18], pneumonia diagnosis on chest X-ray [17], and tuberculosis diagnosis on chest X-ray [17-18]. Another rapidly developing area of research is the use of DL to diagnose coronavirus disease 2019 (COVID-19). A recent review identified 13 studies utilizing DL to diagnose COVID-19 on imaging (nine studies based on chest computed tomography, four on chest X-ray findings). The area under curve (AUC) of DL algorithms are generally in the 0.85 range, with sensitivity in the 90% range and specificity in the 95% range [19].

Outside of the field of radiology, active AI research has also been conducted in echocardiography. Significant progress has been made in using DL to guide image acquisition, measure ejection fraction, measure wall thickness, detect wall motion abnormalities, assess ventricular function, and assess valvular function. DL has also been applied to help make more advanced diagnoses including heart failure with preserved ejection fraction, cardiomyopathy, amyloidosis, and pulmonary hypertension [20-24].

Point-of-care ultrasound (POCUS) is the mode of medical imaging most directly utilized by emergency physicians in practice. Though the potential of DL in POCUS has yet to be fully explored, a few promising research studies have recently been published. Blaivas et al. created a CNN using publicly available software tools and trained it to classify seven common POCUS exam types: pelvis, heart, lung, abdomen, musculoskeletal, ocular, and central vascular access. They then used the algorithm to classify 160 new POCUS clips and compared their accuracy to three blinded POCUS experts. The algorithm accurately classified 70% of new images. The experts correctly classified 93%, 94%, and 98% of images. The time to classification for the algorithm was seven minutes 45 seconds. The three expert reviewers took 27, 32, and 45 minutes to classify the images [25]. In another study, a DL algorithm was created to identify blood vessels, bones, tendons, and nerves on transverse upper extremity POCUS images to aid with landmark training for peripheral vascular access for novices. Its accuracy was then compared to two blinded experts on 50 new POCUS clips. The DL algorithm achieved an AUC of 0.78 versus AUCs of 0.69 and 0.71 for the experts. In labeling blood vessels, only one of the POCUS experts attained an AUC of 0.85, just ahead of the DL algorithm, with an AUC of 0.83 [26]. In a third study, Blaivas et al. created a DL algorithm to analyze collapsibility of the inferior vena cava to predict fluid responsiveness in critically ill patients. The DL algorithm predicted fluid responsiveness with an AUC of 0.70, compared to an AUC of 0.94 by two POCUS experts using video review and manual caliper measurements [27].

Imaging interpretation has been the feature of deep learning [2]. AI applications could potentially revolutionize POCUS by overcoming one of the major obstacles in its widespread use: operator dependence. Algorithms developed using DL could identify landmarks and help guide image acquisition for users of all levels. DL might also provide a quick measurement of key parameters to help guide clinical decision-making in real-time. Such capabilities would be particularly helpful in remote, austere, or mass casualty environments. From the POCUS director’s perspective, DL could be utilized to quickly sort recorded images for quality assurance and credentialing purposes, provide basic POCUS skill instructions for learners of all levels with immediate feedback, and even assess learner competency by comparing scanned images to predefined standards.

Natural language process (NLP) in the EM setting

NLP can be understood as a group of AI and computer science techniques dedicated to process and analyze human natural language data [28]. Natural language represents the communication structure as regularly used by people such as written text or spoken language. In its raw format, such a type of language is not processable by machines. Thus, NLP uses AI algorithms to process text or audio from unstructured sources, extract information, and convert it into machine-processable or structured data. After this conversion, the data from the raw unstructured sources can be used as input to train ML models.

From an EM perspective, NLP has been used to mainly process data registered in the EHR. For example, an NLP approach has supported speech recognition software and templates to help register data in the EHR [2]. Recent studies have used NLP to identify diseases and conditions that are difficult to diagnose by clinical gestalt alone [29]. NLP can help unlock the full potential of EHR data to automatically transform free-text data text from EHR into structured clinical data that can guide diagnostic evaluation and medical decisions. Through the analysis of the information registered in the EHR, NLP approaches have been capable of combining multiple data sources, helping professionals improve diagnostic capacity, specifically for anorexia nervosa, aneurysms, coronary artery disease, Kawasaki disease [30], determination of treatment appropriateness for motor vehicle injuries [31], identification of syncope [32], and prediction of emergency department hospital admission [33] and intensive care unit admission [34].

The use of NLP methods shows promise for enabling the automated extraction of procedural indication data and timeline summarization. The ability to instantly analyze thousands of combinations in terms of procedures performed, lab tests, and diagnosis and associate them with outcomes demonstrates how NLP can affect the quality of care in the EM setting.

Geographic information systems (GIS) and EM response

Health intelligence can be understood as using AI and data science methods and tools to provide accurate, efficient, and productive insights into healthcare and medicine [35]. The recent increase in the computing capabilities of personal machines has allowed the expansion of methods based on AI to address real-world problems. Among multiple possibilities of using AI, the use of algorithms in the geospatial domain is a promising field. Geospatial AI (GeoAI) combines methods in spatial science (e.g., geographic information systems or GIS), AI, data mining, and high-performance computing to extract meaningful knowledge from spatial big data [36-37].

GeoAI applications have used novel sources of spatial big data, such as social media, EHR, satellite remote sensing, and personal sensors, to advance public health science and potentially precision medicine [37]. Using innovative approaches and datasets, GeoAI is creating new opportunities to answer new emerging questions comprehensively.

In EM, GeoAI has fostered the creation of cost-effective solutions to tackle challenges regarding access to emergency services, restructuring of healthcare networks [38], creation of catchment areas [39], estimation of population coverage using satellite imagery [40], identification of mobility patterns [41], and identification of risk areas for specific health events in terms of their characteristics [42].

**Figure 3:**
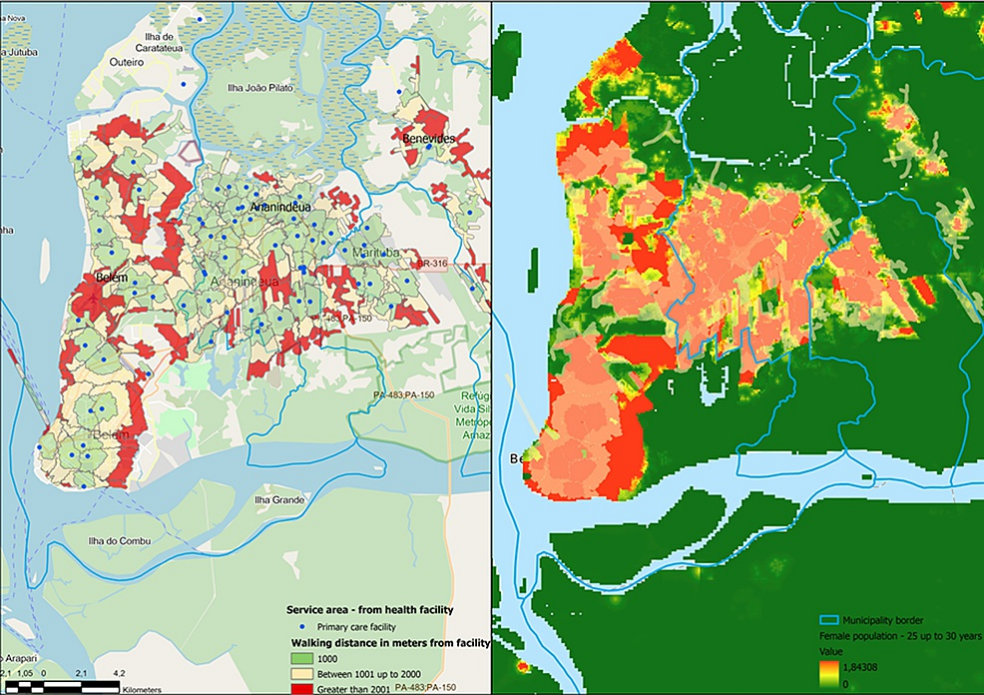
Estimate of population covered by primary care facilities using customized catchment areas and dasymetric population obtained from satellite imagery

Delays in care stemming from large geographic distances from adequately resourced emergency facilities are associated with different levels of outcomes (Figure 3). The use of GeoAI can help policymakers and researchers identify regions facing a lack of emergency care access and support efforts for health system restructuring, resource allocation, and increasing accessibility.

The integration of GeoAI solutions with big data, mobile health, or other available social media data sources can leverage the current possibilities by identifying mobility patterns conjointly with health indicators or outcomes [43]. As the safety net for our community, emergency physicians are in a unique position to identify and solve access to care challenges, our clinical expertise along with GeoAI solutions can inform health system design to optimize referral facility location, inform the best routing strategy to reach underserved areas, and identify populations at more risk for specific diseases [35].

Precision medicine and -omics research

Precision medicine refers broadly to the concept of providing treatments based on a highly individualized analysis of a patient. Typically, when this term is used, it implies the use of in-depth data obtained from or about the patient. Often this includes “-omics” analyses or systematic molecular phenotyping. The overall concept is that the diagnosis alone doesn’t drive treatment decisions but individualized data from the patient guide treatment [44].

There are several levels of molecules that a researcher can now completely analyze from a single patient sample. The suffix “-ome” has come to mean the entire set of molecules that can vary between individuals, and thus “genome” studies the differences in specific genes, proteomics studies the range of proteins, and so on. An example of just a few such modalities includes genomics, transcriptomics, or metabolomics. Additionally, the prefix “pharmaco-” indicates the field of study in which a specific medication may be tailored for use based on an -omic analysis of the patient. The most common form of this is pharmacogenomics. Any of these approaches can produce thousands of data points on a patient from a single biological specimen, such as a peripheral blood sample [45]. Accordingly, ML and AI approaches can be an ideal method for determining which of these data points are associated with disease states of interest.

Increasingly, -omics research is affecting our understanding of emergency medical conditions such as stroke, intracranial hemorrhage, venous thromboembolism, acute coronary syndrome, trauma, respiratory infections, and sepsis [46-53]. This research can take many forms: 1) -omics is a method for efficiently screening for blood-based biomarkers of illness; 2) they can also inform our understanding of the underlying diseases. As an example, combining metabolomics with clinical variables created a more accurate predictor of death from sepsis than clinical and hemodynamic variables alone [54]. As another example, metabolomics had demonstrated the key role of metabolites such as medium-chain fatty acids in predicting risk for acute coronary syndrome [55].

Potential challenges and pitfalls

One of the major challenges to bringing this work from the computer science laboratory to the bedside will be creating the interfaces necessary to implement it on the messy data of the real-time EHR. A review of the 20 most recently published studies cited by Kirubaranjan’s scoping review of AI in EM found that only one was implemented in the EHR, the rest performed on retrospective datasets exported for research [5]. As the work of EHR integration and data cleaning is as great or greater than writing ML algorithms, there is still a large gap between many published academic projects and their readiness for use in clinical practice.

Although ML research can overcome several limitations related to bias, it shares issues that are common to any quantitative research. Foremost, the quality of data tends to add more noise to research analysis, so investigators and consumers need to understand the validity of the data presented. Some models, such as DL, are very flexible and can lead to overfitting, which is a universal issue to any predictive model study. ML researchers will need to understand the pros and cons of each algorithm to address the key question [56]. Lastly, precision medicine leads to a subgroup of patients where targeted intervention can develop, but this also causes privacy issues, which AI is yet to address.

## Conclusions

The timing of ML in data science and precision medicine is ripe now. It has become a part of common research and practice in healthcare, and the community of ML users will continue to grow in the EM community. AI can aid clinical decisions, such as triage decisions, evaluation, treatment, and the diagnostic process, by interpreting images. It can help identify the healthcare gap through GIS and identify a rare disease from text data through NLP. In addition, precision medicine can help elucidate an important biomarker for the condition of interest to EM physicians. Because of computational needs and a deep understanding of bias and variance related to data science, interested EM researchers need to establish a strong alliance with partners with additional skill sets in computational science. Another area of development is that AI researchers are developing more interpretable models from unsupervised models, creating semi-supervised ML. We need to see more studies comparing AI and “standard of care” or clinical judgments. In the near future, you may see more AI taking on tasks that were once operated by healthcare workers, thus achieving harmonization.
